# PlayMolecule Glimpse: Understanding Protein–Ligand Property Predictions with Interpretable Neural Networks

**DOI:** 10.1021/acs.jcim.1c00691

**Published:** 2022-01-03

**Authors:** Alejandro Varela-Rial, Iain Maryanow, Maciej Majewski, Stefan Doerr, Nikolai Schapin, José Jiménez-Luna, Gianni De Fabritiis

**Affiliations:** † Computational Science Laboratory, Universitat Pompeu Fabra, Barcelona Biomedical Research Park (PRBB), Carrer Dr. Aiguader 88, 08003 Barcelona, Spain; ‡ Acellera Labs, Doctor Trueta 183, 08005 Barcelona, Spain; ¶ Institució Catalana de Recerca i Estudis Avançats (ICREA), Passeig Lluis Companys 23, 08010 Barcelona, Spain

## Abstract

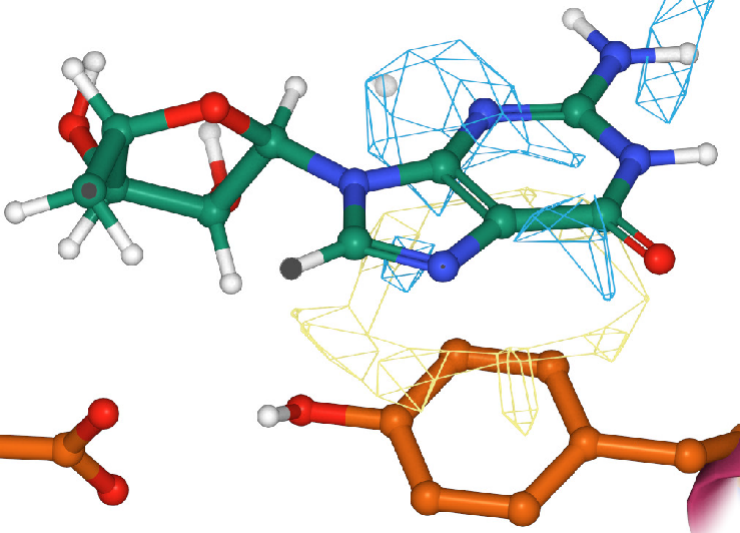

Deep learning has been successfully applied to structure-based protein–ligand affinity prediction, yet the black box nature of these models raises some questions. In a previous study, we presented K_DEEP_, a convolutional neural network that predicted the binding affinity of a given protein–ligand complex while reaching state-of-the-art performance. However, it was unclear what this model was learning. In this work, we present a new application to visualize the contribution of each input atom to the prediction made by the convolutional neural network, aiding in the interpretability of such predictions. The results suggest that K_DEEP_ is able to learn meaningful chemistry signals from the data, but it has also exposed the inaccuracies of the current model, serving as a guideline for further optimization of our prediction tools.

## Introduction

Machine-learning methods have been widely applied in the field of chemoinformatics, ranging from simple, regressor-based QSAR models^1−4^ to more complex neural networks. These latter methods have been reported to increase performance in some critical tasks for drug discovery, such as toxicity assessment,^5,6^ pharmacokinetics, physicochemical property prediction,^7−10^ and protein–ligand binding affinity prediction.^11−15^

In a previous work, we developed K_DEEP_–a 3D convolutional neural network (CNN) that accepts as input a voxelized representation of a protein–ligand complex and outputs a prediction of binding affinity with state-of-the-art accuracy.^11^ However, it was unclear whether K_DEEP_ was learning meaningful chemistry or just exploiting shortcuts such as the positive relationship between molecular weight and affinity.^16^ Learning these shortcuts instead of the underlying nature of the problem is a topic of concern in the field.^17^ It is then comprehensible for many machine learning methods to spark criticism regarding the difficulty to understand the rationale behind their predictions. It has been questioned whether a pharmaceutical company would promote a given molecule into a portfolio based only on an opaque prediction made by a neural network, without any clear explanation to support it.^18^ Providing such an explanation would undoubtedly increase the value, trustworthiness, and usability of machine learning models in drug discovery.

Recently, advances in model interpretability,^19,20^ as well as the availability of software libraries such as Captum^21^ and Alibi,^22^ have allowed researchers to get a first glimpse of what features of the input are more influential toward predictions made by neural networks (i.e., feature attribution assignment). One natural approach to measure this influence is to look at the gradients of the output neuron with respect to the input. In fact, in a CNN trained to discriminate accurate from inaccurate binding poses and to predict binding affinity, visually inspecting these gradients can reveal in which direction the atoms should move to improve the score that the network assigns it,^23^ providing some degree of interpretability.

However, backpropagating the prediction relative to the input layer can produce very low gradients in the vicinity of the input vector,^19^ a process which is known as gradient saturation. The Integrated Gradients (IG) feature attribution technique^19^ helps to mitigate this problem, providing a better measure of how each input feature influences the prediction. Instead of evaluating the gradients at one particular input value (the image in a traditional 2D-CNN), gradients are computed for several variants of that image, ranging from a user-defined baseline (typically, an image with all its pixel-channel values set to zero) to the actual image. In each variant, the values of all its pixels are multiplied by a scalar α, ranging from the zeroed-out input to the original image. At low values of α, the resulting input vector is far from the usual input space the network has been exposed to during training, circumventing the gradient saturation issue.

In this article, we present an application to visualize the contribution of the input features for the prediction of K_DEEP_ and similar CNNs. In addition to describing the methodology used herein, we also showcase several relevant examples of attributions which match with structural biology knowledge. We analyze the prediction of three distinct models: a clash detector, a docking pose classifier, and K_DEEP_. The clash detector provides a baseline to which we compare the other models and allowed us to validate the implementation of this application. The docking pose classifier and K_DEEP_ models were evaluated to see if CNNs trained to perform chemically relevant tasks were learning meaningful chemistry. The application, called Glimpse, is available to use at https://www.playmolecule.org/Glimpse/.

## Methods

### Model Training

K_DEEP_ is a 3D-CNN which accepts as input a grid of size . This grid is generated by mapping the atom positions of the ligand and its surrounding protein residues to the corresponding voxel and channel in the grid. K_DEEP_ uses 8 different channels: hydrophobic, aromatic, hydrogen bond donor, hydrogen bond acceptor, positive ionizable, negative ionizable, metals, and excluded volume (occupancy) for both protein and ligand. This gives a total of 16 channels and a grid of dimensions  (see Jiménez et al.^11,24^ for more details). The network was trained on the latest version of the refined set of PDBbind,^25^ achieving a Pearson’s correlation coefficient of 0.79 in the test set. Details on the training and evaluation of the different models can be found in the SI.

We also trained a clash detector. The objective behind it is two-fold. First, there is a clear expectation in terms of what attributions should look like: clashing regions or close contacts would appear highlighted, while the remaining voxels would be of little importance. We were able to validate the implementation of Glimpse by checking if the computed attributions matched this expectation. Second, the computed attributions of this simple model served as a reference point to which we compare the other models, both visually and quantitatively. This model was trained to discriminate regular protein–ligand poses from clashed poses and achieved 0.97 classification accuracy and 0.98 precision in a held-out validation set.

We trained a third model–a docking pose classifier–for two reasons: (i) it is a challenging task, comparable to that of predicting binding affinity, and (ii) there is much more data available from which the model can learn. This model was trained on a large set of good (RMSD  Å) and bad poses (RMSD  Å), showing an accuracy and precision of 0.94 and 0.83, respectively, on the validation set.

It must be noted that the performance of these three models was evaluated on a random test and validation sets. In some cases, protein–ligand complexes in these sets might be similar to those in the training set, either in terms of protein structure or ligand composition. This yields overoptimistic results. In fact, when trained and evaluated in more strict splits which ensured sequence and ligand dissimilarity, the K_DEEP_ performance ranged from *r* = 0.09 to *r* = 0.7 (see the SI for details).

The models analyzed in this work have been uploaded to Glimpse with the following names: “K_DEEP_”, “Pose classifier”, and “Clash detector”. Attributions for these models can be computed and visualized in the app for any valid protein–ligand complex.

## Implementation

### Integrated Gradients

The IG method works by computing gradients of the output neuron with respect to the input layer along an interpolated path from a given input baseline () to the original input (*x*_*i*_) taking α infinitesimal steps as in^19^
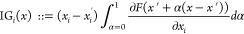
1where *F* denotes the forward pass of the neural network.

This effectively circumvents the issue of low gradients (gradient saturation) in the vicinity of the input by averaging the gradients along a range of different input values. Gradient saturation can occur if a given input value leads to a neuron being activated in a region of the activation function which is very flat, for instance, the extremes of a sigmoid. Hence, using the gradients of the prediction with respect to a given input could assign an importance of zero to it, regardless of its real importance.

The computed attributions provide a value for each voxel representing their importance toward the prediction. Glimpse uses the IG implementation from the Captum library.^21^ Attributions are computed by approximating the integral as a series of discrete steps along the interpolated path from the selected baseline (an input vector in which all voxels are set to zero) to the evaluated, voxel map corresponding to the original protein–ligand complex. In this implementation, we used 100 steps, as it was shown to offer a good balance between computational expense and attribution quality.

### Graphical User Interface

**Figure 1:**
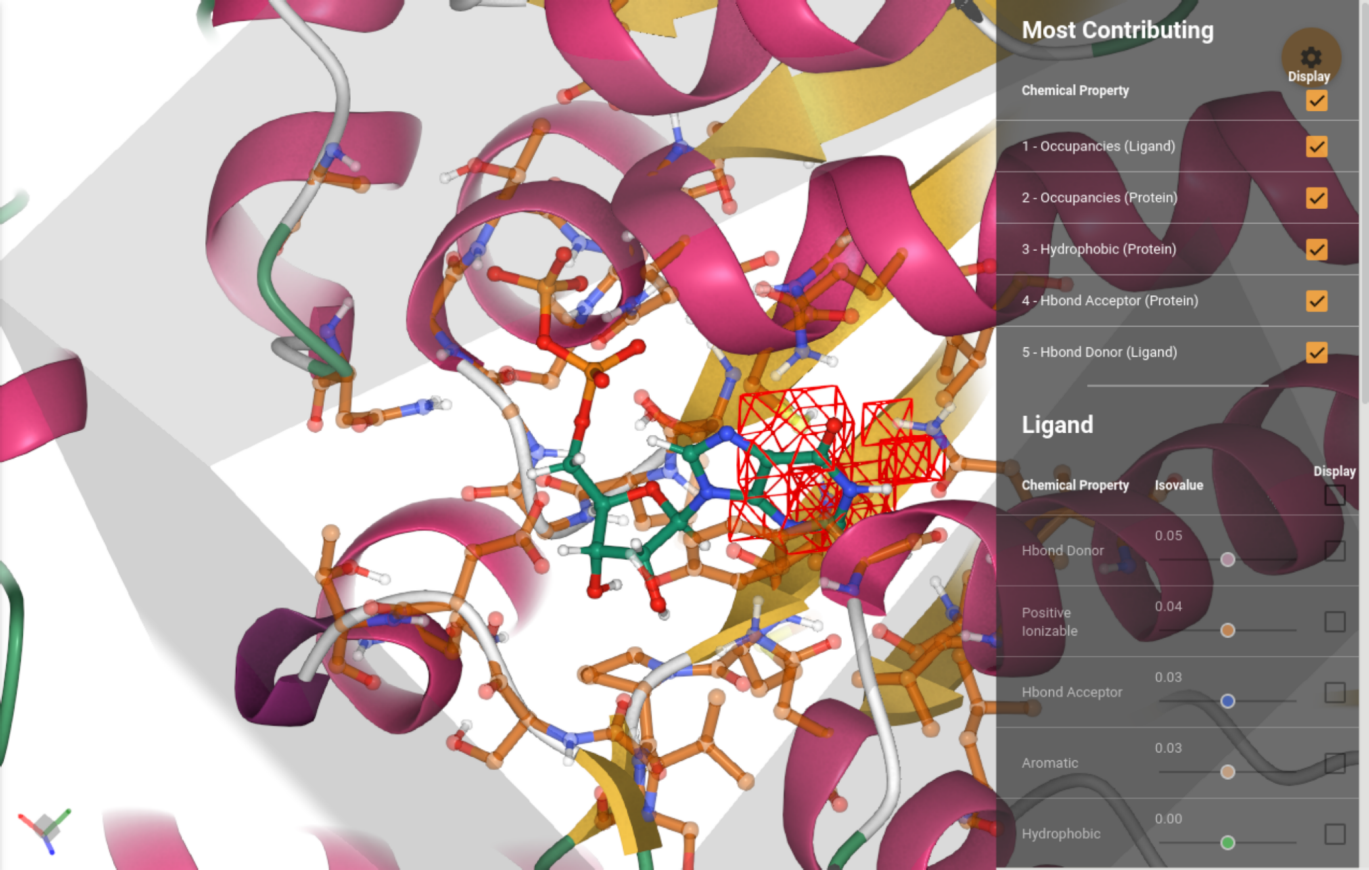
Main view of the graphical user interface. The protein–ligand complex is displayed, with the attributions of the most contributing voxels superimposed. The attributions for the different channels can be seen individually using the corresponding sliders in the menu on the right, which display isosurfaces at different isovalues. The full protein is shown in a cartoon representation, while residues in the binding site (defined by being within 5 Å to the ligand) are shown in a transparent ball–stick representation (only heavy atoms and polar hydrogens). The all-atom representation of the ligand is shown in a bold ball–stick. The region of space seen by the model (voxelization cube) is delimited by a transparent, gray box.

Glimpse provides a web-based graphical user interface (GUI) that helps to trace attributions to voxel maps. An overview of the GUI is provided in Figure 1. The computed attributions for the input channels are displayed as mesh isosurfaces whose isovalue can be tuned with a slider. A detailed description of the input channels is provided in the Model Training section of this manuscript. To offer a summary of the results, the interface displays by default only the most contributing regions. These regions are the result of identifying, for each of the 16 channels, the voxel with the highest absolute attribution value and the neighbors around it. In this summary, only the channels containing the best 5 voxels are displayed for simplicity. Additionally, the user can display the attributions for the different input channels individually, and the raw attribution maps can be downloaded as a Gaussian cube format file (.cube) and explored in VMD^26^ or other molecular visualization software.

### Usage

Glimpse requires a protein–ligand complex structure, either experimentally determined or predicted by a docking software. The protein must be correctly protonated and provided as a .PDB file. The PlayMolecule platform offers proteinPrepare^27^ to protonate the protein. The ligands have to be provided a valid SDF file. If needed, Glimpse provides an option for protonation of these ligands. Only 100 ligands are allowed per job. Finally, users can select which model to use from a list, which by default is “K_DEEP_”. In terms of time, evaluating 10 protein–ligand complexes takes around 150 s. When inspecting the attributions, one would typically start looking at the visual summary, followed by an inspection of individual channels. It is worth paying particular attention to the voxels with the highest and lowest attribution values and checking if the nearby atoms are involved in an interaction. The occupancy channels offer a good overview of the whole picture and constitute a good place to start.

### Analysis

For each model, we visually inspected the attributions computed for several protein–ligand complexes to evaluate how well they match with structural biology knowledge. We focused on interactions known to contribute toward binding free energy, e.g., hydrogen bonds or π-stacking. Another aspect we inspected was the reciprocity in the attributions, that is, whether the two parties involved in the protein–ligand interaction are reflected in their attribution values.

While visual inspection can provide valuable insights, it can also be misleading and prone to unintended biases. Therefore, we designed a quantitative analysis, in which we computed the IG for all the crystal structures (not clashed or docked) coming from the PDBbind database. For each of the 16 channels, the voxel with the highest, absolute value of the IG values was identified. Then, we measured the distance between the top contributing voxels in the complementary channels. We evaluated the following pairs of channels: protein hydrophobic and ligand hydrophobic, protein aromatic and ligand aromatic (π-stacking), protein acceptor and ligand donor, protein donor and ligand acceptor (hydrogen bonds), protein occupancy and ligand occupancy (steric component). As a baseline, we took the distance between two randomly selected voxels from the appropriate channels, whose occupancy value was over 0.75, ensuring that an atom was nearby the voxel.

## Results

As a leading example for the analysis, we selected a complex of a molecular chaperone, heat shock protein 90 kDa (Hsp90) sourced from the PDB (PDBid: 3D0B).^28^ This well studied oncology target has been a subject of numerous structure based virtual screening campaigns.^29,30^ In the analyzed example, HSP90 forms a potent complex with an analogue of benzamide tetrahydro-4H-carbazol-4-one (SNX), with an affinity of 290 nM. Additionally, the complex possesses few features that facilitate tight binding, mainly π-stacking and a hydrogen bond with a conserved aspartate (D93)–a very frequent interaction among HSP90 inhibitors.^31^ This helped us to relate the predictions to structural features of the complex.

### Clash Detector

As a sanity check, we started by evaluating the attributions of the simplest model, the clash detector. The visual inspection of multiple complexes revealed that, in all inspected cases, clashing regions or close contacts were highlighted, while residues far apart from the ligand remained ignored. The clashes were clearly indicated by occupancy channels of both protein and ligand, showing reciprocity (Figure 2.1A). An example of HSP90 with a clashed pose clearly highlights the clashed region between the ligand and a leucine (L92) in the pocket (Figure 2.1B). This confirmed that the protocol was working correctly and gave us a baseline for the analysis of the following models.

**Figure 2:**
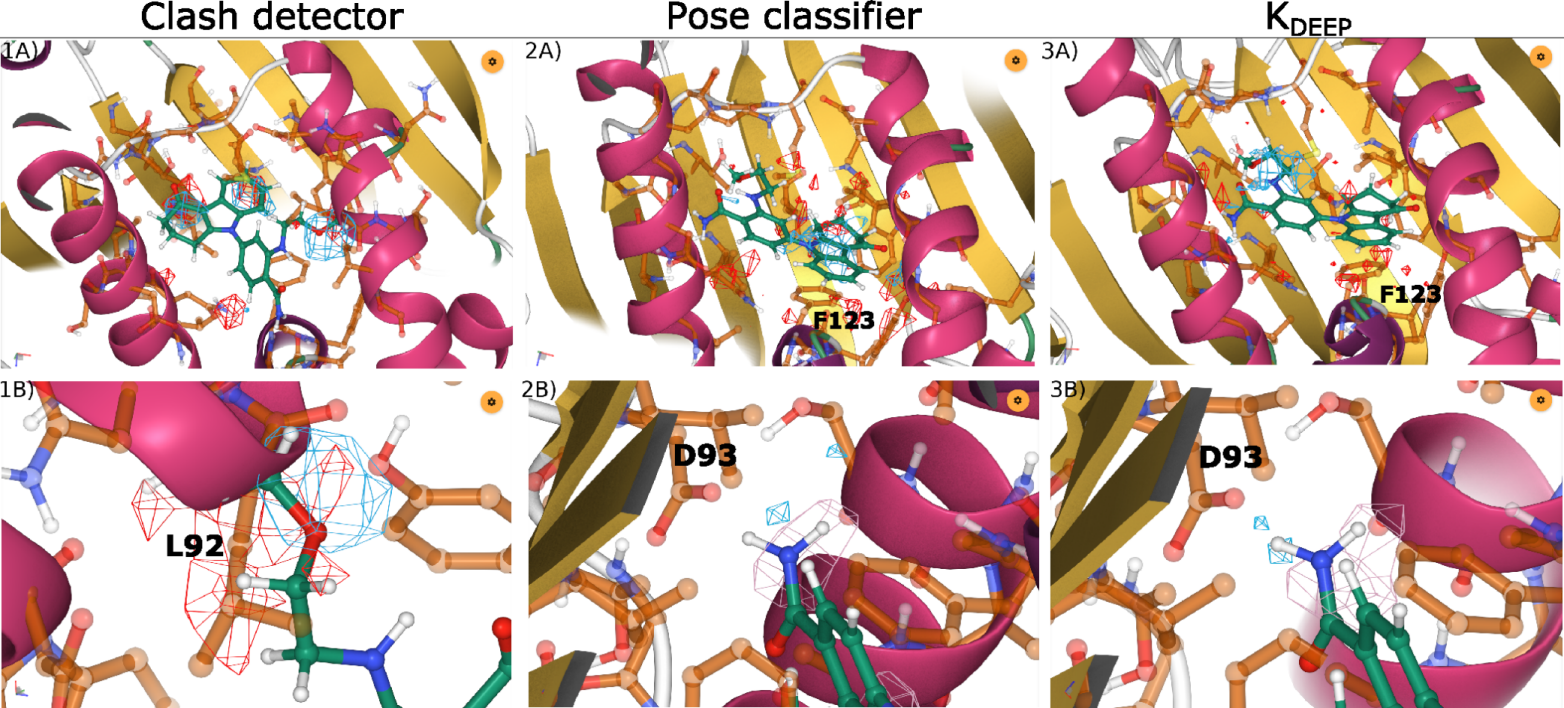
Comparison of computed attributions obtained for a complex of HSP90 with an analogue of benzamide tetrahydro-4H-carbazol-4-one (PDB code: 3D0B) by the three models: Clash detector (1A and 1B), Pose classifier (2A and 2B), and K_DEEP_ (3A and 3B). Pictures on the top row show the attributions for the protein and ligand occupancy channels, in red and blue, respectively. The bottom row focuses on particular interactions. 1B shows a clash between the ligand and the leucine and the attributions for the occupancy channels of protein and ligand (red and blue). 2B and 3B show the hydrogen bond between the benzamide moiety in the ligand and the aspartate (D93) residue in the protein. Attributions for the ligand donor channel are shown in pink, while for the protein acceptor channel, the attributions are shown in blue.

### Docking Pose Classifier

The next model was trained to discriminate good and bad docking poses. The true binding mode depends on an enthalpic factor which is determined by formation of strong and stable interactions between the ligand and the protein, like hydrogen bonds or π-stacking.^32^ Hence, we expect models trained to perform such tasks to have learned these interactions.

We found several examples where the attributions correctly matched these expectations. Figure 2.2B shows a hydrogen bond being highlighted in the appropriate channels: hydrogen bond acceptor in the protein and donor in the ligand. An amide moiety in the ligand is establishing a hydrogen bond with the nearby aspartate (D93). It is indeed a key interaction frequently featured in HSP90 inhibitors. In addition to this hydrogen bond, a π-stacking interaction takes place between the aromatic ring of the ligand and a phenylalanine (F123) in the protein, as highlighted by the attributions for the occupancy (Figure 2.2A) and aromatic channels (not shown) of both the protein and the ligand.

After inspecting several examples, we saw that, as in the clash detector model, residues far from the ligand were ignored for the most part (Figure S7 shows one exception), and reciprocity between the ligand and the protein atoms was observed in the majority of inspected cases. However, the overall picture was less clear than in the clash detector model as can be seen by comparing the global view of the three models (sections 1A, 2A, and 3A of Figure 2). While in the clash detector model high attributions are well focused on the clashing regions and close contacts, the other two models exhibit a more disperse view.

### K_DEEP_

Similarly to the previous model, K_DEEP_ is expected to predict binding affinity by detecting and correctly weighting the molecular interactions between protein and ligand. In the majority of inspected cases, we saw reciprocity between the ligand and the protein atoms that formed interactions (Figure 2.3A), while protein residues distant from the ligand remained ignored (Figure S8 shows one exception). The overall attribution maps seem to be more disperse than for docking pose predictor.

For the example of HSP90, the predicted affinity value was 75 nM, reasonably close to the experimental value of 290 nM, making it a suitable example for attribution analysis. As in the case of the pose classifier, the network correctly identified the key hydrogen bond with D93 (Figure 2.3B), as well as π-stacking between the aromatic ring of the ligand and F123 (Figure 2.3A). In this case, however, only the phenylalanine ring is highlighted. Nonetheless, the attributions of the aromatic channels highlight the aromatic residues in close proximity to the ring system of the ligand, including Phe, ignoring all the other aromatic residues in the box.

### Quantitative Analysis

**Figure 3:**
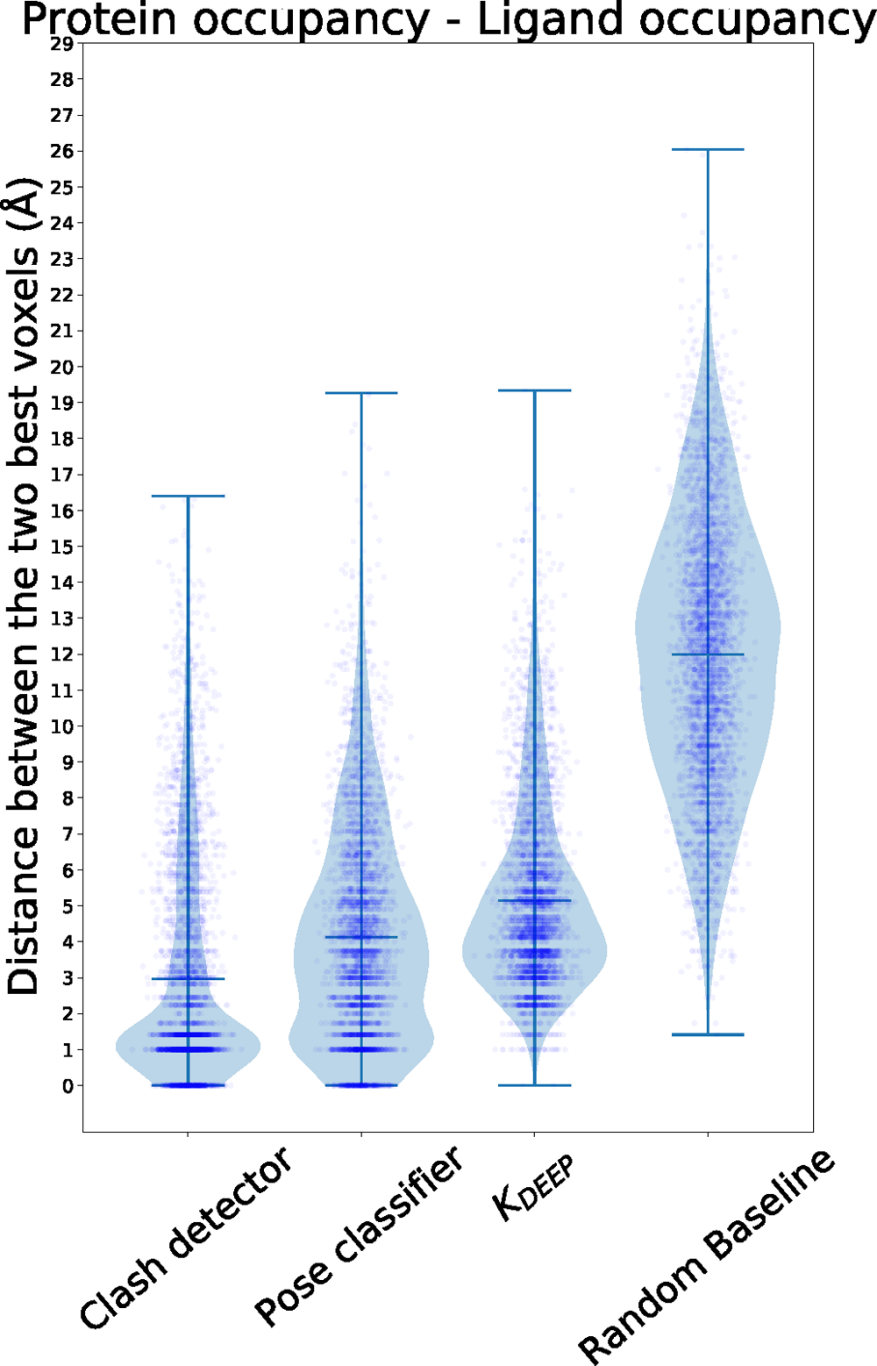
Distance distribution between the two voxels with the highest, absolute value in protein and ligand occupancy channels.

The quantitative analysis confirmed, for the most part, the conclusions obtained by the visual inspection. Figure 3 shows the distribution of distances between the top voxels from protein occupancy and ligand occupancy channels. Figures S1–S6 show the distance distribution for the remaining relevant combinations of channels. We can see that models have learned that ligand and protein atoms close to each other are important, which is exemplified by the different distributions being shifted toward the contact range ( Å). This hints that the networks are learning relevant features of the complex: close contacts in the case of clash detector and interactions for the two remaining models. This is particularly clear for the clash detector, where the distance distribution is radically shifted toward the range under 3 Å. The pose classifier model follows and shows a similar, shifted distribution, although not as clear as in the clash detector. In these two models, the cloud of points describes a 3-line pattern at 0 and 1 and around 1.5 Å, showing that the most contributing voxels were in very close proximity. In fact, the high number of examples observed at 0 Å distance indicates that, in a large fraction of complexes, the same voxel in the two relevant channels was the most highlighted. The distance distribution for K_DEEP_ is slightly shifted toward higher values but is still much better than the random baseline. This difference could be related to the fact that, during training, K_DEEP_ is only exposed to crystal poses, in which the distances between ligand and protein atoms should be uniform across examples. This is obviously not the case in the clash detector nor in the pose classifier, as docking might generate poses which, in some areas, might be slightly too close or too far away from the surface, which might correlate with a bad pose. Hence, these two models would benefit more from paying attention to this low range of values, while K_DEEP_ might not. A similar scenario occurs to the other combinations of channels (Figures S1–S6): The clash detector is usually the best, followed closely by the pose classifier and K_DEEP_, which show similar distributions. The distributions of all three models are significantly different from the random baseline in all channel combinations studied according to a two-sided Mann–Whitney U Test (all combinations had a *p*-value lower than the significance threshold 0.0001). These results provide evidence supporting the hypothesis that K_DEEP_ and the pose classifier models have learned to focus on the interface between the ligand and the protein, as a trained chemist or biologist would do.

After this test, we checked if there was any correlation between the magnitude of the attribution of the two best voxels and the distance between them as a means to test if the quality of the explanation (reciprocity between the two parties) was correlated with the magnitude of the attribution. As in the prior experiment, we identified the voxels with the highest attribution values in the same pairs of channels and annotated the distance between them. We then evaluated the correlation between the sum of the attributions in those two voxels and their distance. For all models and combinations of channels studied, the Pearson’s correlation coefficient ranged between −0.48 and −0.15 (Figure S9). Hence, the higher the attribution value of the two voxels, the more likely it is that those two voxels are in proximity.

Furthermore, for the K_DEEP_ model, we also evaluated if there was a correlation between the attribution values and the accuracy of the prediction (measured as an absolute difference between predicted and actual p*K*_D_ values). Neither the maximum attribution value across all channels nor the sum of the absolute attribution values correlated with the accuracy of the prediction in the K_DEEP_ model (Pearson’s *r* was −0.05 and −0.02, respectively). There was not any strong correlation either between far away residues being highlighted and prediction accuracy (*r* = 0.05, see the SI for details).

Finally, we evaluated how sensitive the attributions were to minor changes in the input, namely (1) rotations of the complex and (2) slight modifications of the protein–ligand pose. Ideally, the attributions should be consistent across different orientations and pose variants; hence, the same atoms should be highlighted in the different variations. All three models show a greater consistency than the expected by random, both for protein and ligand atoms. Although on average, the same atom was selected just around 2 times out of the 10 input variants (random baseline is close to 1.0); in all three models, in a great fraction of complexes, the same atom is picked more than 4 times, which is not the case in the random baseline (Figures S10–S13). These results show that the attributions tolerate some degree of input variability. Details on these experiments can be found in the SI.

## Conclusion

The results indicate that the trained networks are able to learn meaningful chemical interactions. However, for the pose classifier model and K_DEEP_, some cases were observed where the network had ignored strong interactions, highlighted residues far from the ligand (Figures S7 and S8) or highlighted ligand atoms whose interaction counterpart in the protein had low attributions. This can be the result of the difficulty of associating the occurrence of certain contacts or interactions with affinity or with the quality of the pose prediction, leading to shortcut learning. For instance, if all the complexes for kinases in the training set have a p*K*_d_ of 5.0, the network might learn to identify this family by using a set of characteristic residues (which could be far from the ligand) and simply predict 5.0. In this sense, PDBbind is not an ideal training set, as the total number of examples contains few samples for deep-learning standards, and p*K*_d_ values are distributed in a large range from 2 to 12, scarcely populated in both extremes. In the case of the clash detector model, we have more examples for each class, the two classes belong to very different distributions, and it is very easy to associate the occurrence of a given pattern in the input (a clash) to the correct class, discouraging shortcut learning, which manifests in the attributions of this model being much clearer.

Furthermore, voxelization is limited to only 8 properties and excludes crystallographic waters. Given that the latter are known to mediate certain protein–ligand interactions (e.g., water bridges), a fraction of the variability in the binding affinity can only be explained by the presence of these waters. Additionally, hydrogen-bond donors and acceptors have diverse strengths (thiol being a weak donor and hydroxyl being a strong one); however, in the featurization, they are grouped together in just two entities (donor and acceptor). The same reasoning applies for the positive and negative ionizable channels.

In this study, we have shown that Glimpse displays the capability to expose some of the flaws of the networks herein analyzed, suggesting that it can act as a useful diagnostic tool for structure-based 3D-CNN models. We were also able to identify atoms or regions of the protein–ligand complex that play a bigger role on the predictions made by the networks, which is key to improve the usability of CNNs in computational chemistry.

**Data and Software Availability**. Glimpse is available free of charge at https://www.playmolecule.org/Glimpse/. The three models studied in this article (“K_DEEP_”, “Pose classifier”, and “Clash detector”) can be found and used in the web interface. The protein–ligand complexes we used as input for generating the images are available to download in the “Examples” tab in the web interface. The databases used for training and validating the models (BindingMoad^33^ and PDBbind^25^) are publicly available, as well as the docking software (rDock^34^).

## Supporting Information Available

“Model training” and “quantitative analysis”, additional information for these sections; Figures S1–S6, distance distribution between the two voxels with highest, absolute attribution value for different channel combinations studied; Figures S7 and S8, examples of protein residues far from ligand having high attribution values; Figure S9, correlation between magnitude of attributions of two best voxels and distance between them; and Figures S10–S13, attribution consistency distributions (PDF)
