# Supplementary material for: PlayMolecule Glimpse: Understanding Protein–Ligand Property Predictions with Interpretable Neural Networks
Source: J Chem Inf Model. 2022 Jan 3;62(2):225–31. doi: 10.1021/acs.jcim.1c00691 (PMC8790755; doi:10.1021/acs.jcim.1c00691)
Supplement: Supplementary file 1 — ci1c00691_si_001.pdf [file ci1c00691_si_001.pdf]

Supplementary Information
PlayMolecule Glimpse: Understanding protein-ligand property predictions with interpretable neural networks

Alejandro Varela-Rial,[†,‡] Iain Maryanow,[‡] Maciej Majewski,[†] Stefan Doerr,[‡] Nikolai Schapin,[†,‡] José Jiménez-Luna,[†] and Gianni De Fabritiis[*,†,‡,¶]

†*Computational Science Laboratory, Universitat Pompeu Fabra, Barcelona Biomedical Research Park (PRBB), Carrer Dr. Aiguader 88, 08003, Barcelona, Spain*

‡*Acellera Labs, Doctor Trueta 183, 08005, Barcelona, Spain*

¶*Institució Catalana de Recerca i Estudis Avançats (ICREA), Passeig Lluis Companys 23, 08010 Barcelona, Spain*

E-mail: gianni.defabritiis@upf.edu

Model Training

K_{DEEP}

K_{DEEP} was trained on the latest version of the refined set of PDBbind[1] comprising of 4749 protein-ligand complexes after filtering of duplicates and complexes that failed the preparation. A validation set was created for hyperparameter tuning and early stopping by taking a random sample of 10% of the codes in the refined set. As a test set, the core set of PDBbind was used, which is comprised of 272 complexes.

For all three models, during training and validation, the protein-ligand complex was rotated around the geometrical center of the ligand before generating the final grid, in order to augment the existing training set and compensate for the fact that CNNs are not rotationally invariant.[2]

Clash detector

To train the clash detector model, we used the complexes available in the refined set of the 2019 version of PDBbind. The clashed poses were artificially generated by randomly rotating the ligand on its own geometrical center while ensuring that at least one atom in the ligand was within a distance of 1.5Å to the protein. We used the same architecture as for K_{DEEP}, and the binary cross-entropy loss function.[3] We trained the model for 50 epochs with a batch size of 32 and a starting learning rate of 10^{-3}. This model achieved 0.97 classification accuracy and 0.98 precision in a held-out validation set, constituted by a randomly selected group of 10% of the protein-ligand complexes, for which clashed poses were also generated. Both the training and validation sets were constructed in a balanced way, so that half the examples were crystal poses and the other half were clashed poses.

Pose classifier

The pose classifier model was trained on BindingMoad database,[4] which contains $38,702$ protein-ligand complexes. Ten docked poses were generated for each complex using the rDock docking software,[5] which led to more than $310,110$ examples after removing failed jobs. This set was split into two classes, one featuring "good" poses (poses with RMSD below 1 Å) and "bad" poses (RMSD greater than 3 Å). Poses between 1 and 3 Å were discarded, similar to the work by,[2] to create a greater separation between the two distributions and ease the classification task. The final number of examples was $270,225$, constituting a much larger training set than the other two models. This model was trained with the same hyper-parameters, loss function and architecture as the previous one.

A validation set was created, composed by all the good and bad poses generated for a random selection of 10% of the protein-ligand complexes in the BindingMoad database, so that poses for the same protein-ligand complex cannot be found in both training and validation sets. Because most of the poses belonged to the "bad" category, a sampling correction was introduced in the training and a number of bad poses in the validation were removed to reach a 1:1 ratio, reaching a total of $17,478$ examples in the validation set.

Strict split: K_{DEEP}

For K_{DEEP}, we designed a more strict split, where the PDBbind refined set was clustered by sequence similarity using a 70% threshold. The three biggest clusters were selected for testing. A final filter was applied to these three clusters to discard complexes whose ligands had a fingerprint similarity greater than 0.6 with any ligand in any other cluster ensuring that these test sets were different both in terms of protein sequence and ligand composition from any other cluster. Finally, three different K_{DEEP} models were trained using one of the three clusters as test set (leaving one cluster out and training in all the others). Pearson's correlation coefficient in these three test sets was 0.70 (N=29), 0.28 (N=152) and 0.09 (N=81). Hence, predictive performance is lower than on the less strict split and it might be family-dependent.

Quantitative analysis

Correlation between far away residues and accuracy

We tried to measure if any correlation existed between the presence of far protein residues being highlighted and prediction accuracy. We summed the attributions of all protein channels for the voxels that were further than 8 Åfrom any ligand atom (bad attributions) and divided it by the sum of all protein attributions, obtaining a percentage of the attributions falling far from the ligand. The Pearson's correlation with the prediction error was just 0.05 meaning that the presence of far away residues being highlighted does not correlate well with

prediction accuracy.

Attribution consistency across rotations and pose variations

In order to check how sensitive attributions were to changes in the protein-ligand complex's orientation, attributions were computed for 10 different orientations for each system. Then the ligand and protein atoms closest to the voxel with the highest attribution in the occupancy channels were identified in each rotation. This allowed us to evaluate how consistent was the selection in comparison to a random baseline, where the ligand and protein atoms were selected randomly among those inside the $24\mathring{A}^3$ box. A similar experiment was performed to evaluate attribution's sensitivity to changes in the pose. Here, instead of 10 rotations, we applied minor rotations (up to 12°) and displacements (sampled from a normal distribution with mean 0 and std 0.2Å) to the ligand alone, leading to 10 small variants of the same pose. Due to the computational cost of these experiments, both of them were performed in a random subset of 300 complexes sampled from PDBbind refined set.

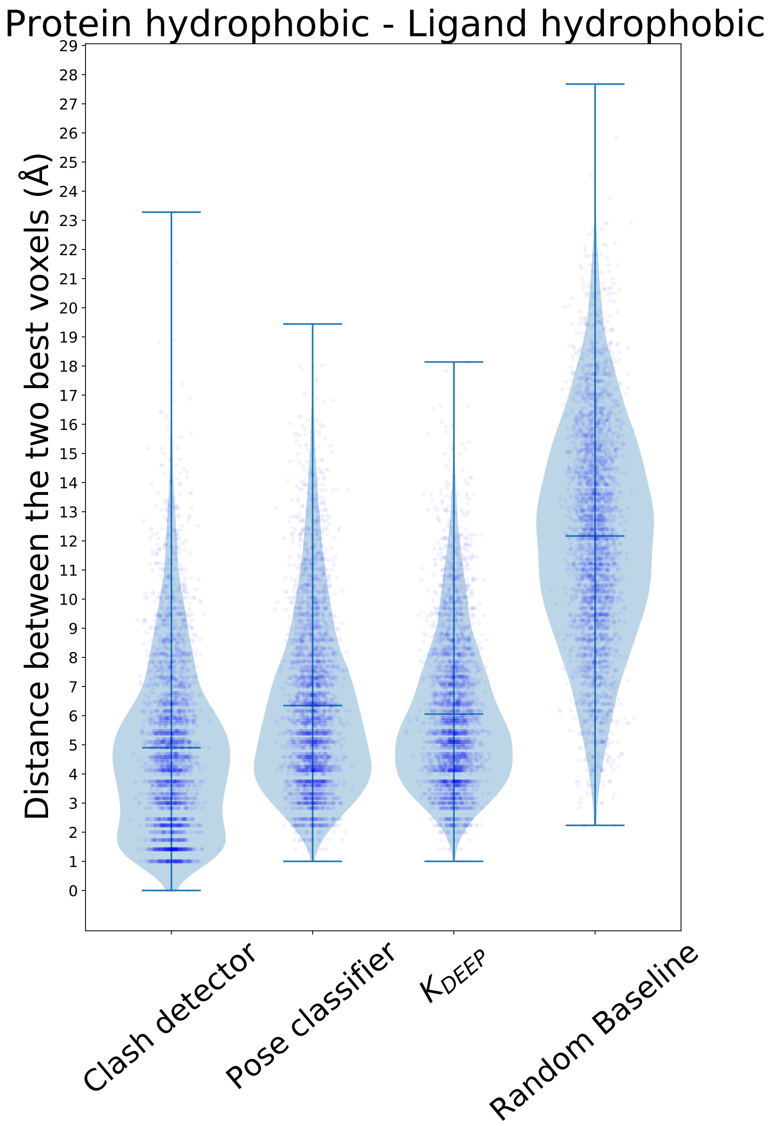

Figure S1: Distance distribution between the two voxels with highest, absolute attribution value in protein hydrophobic and ligand hydrophobic channels.

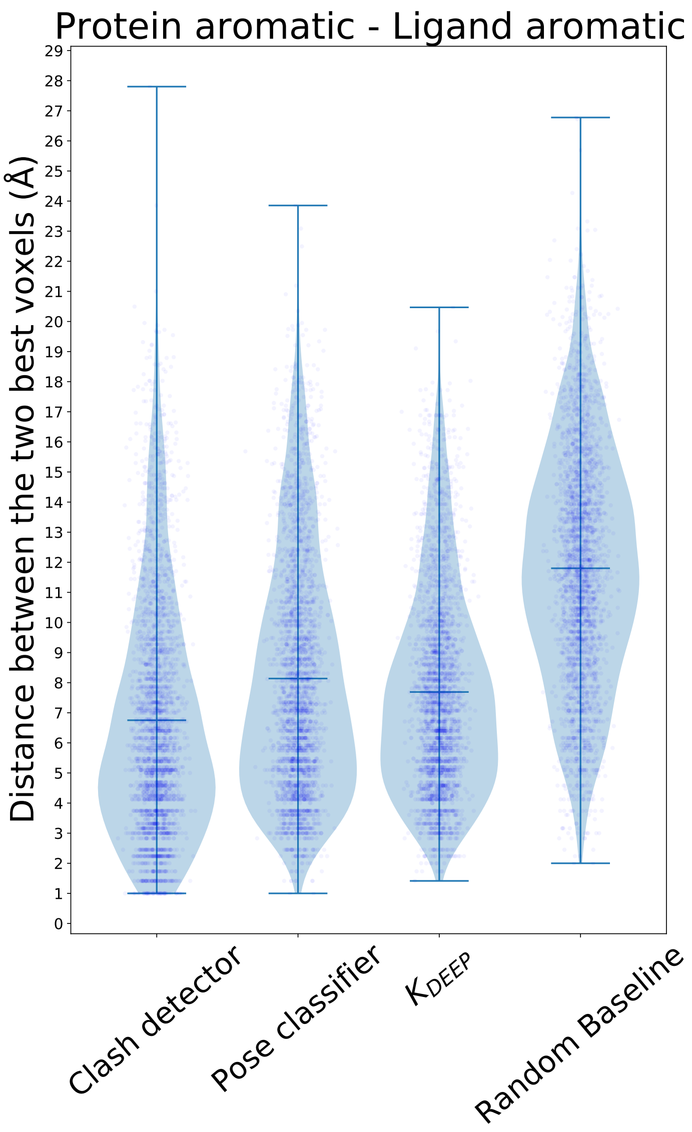

Figure S2: Distance distribution between the two voxels with highest, absolute attribution value in protein aromatic and ligand aromatic channels.

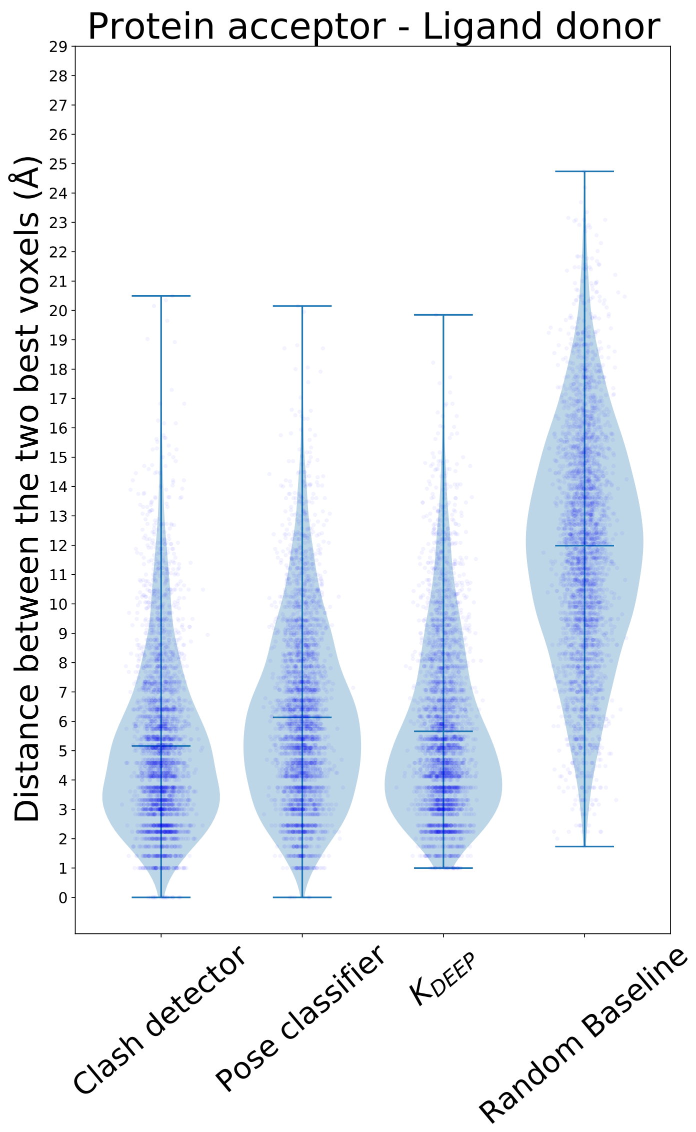

Figure S3: Distance distribution between the two voxels with highest, absolute attribution value in protein acceptor and ligand donor channels.

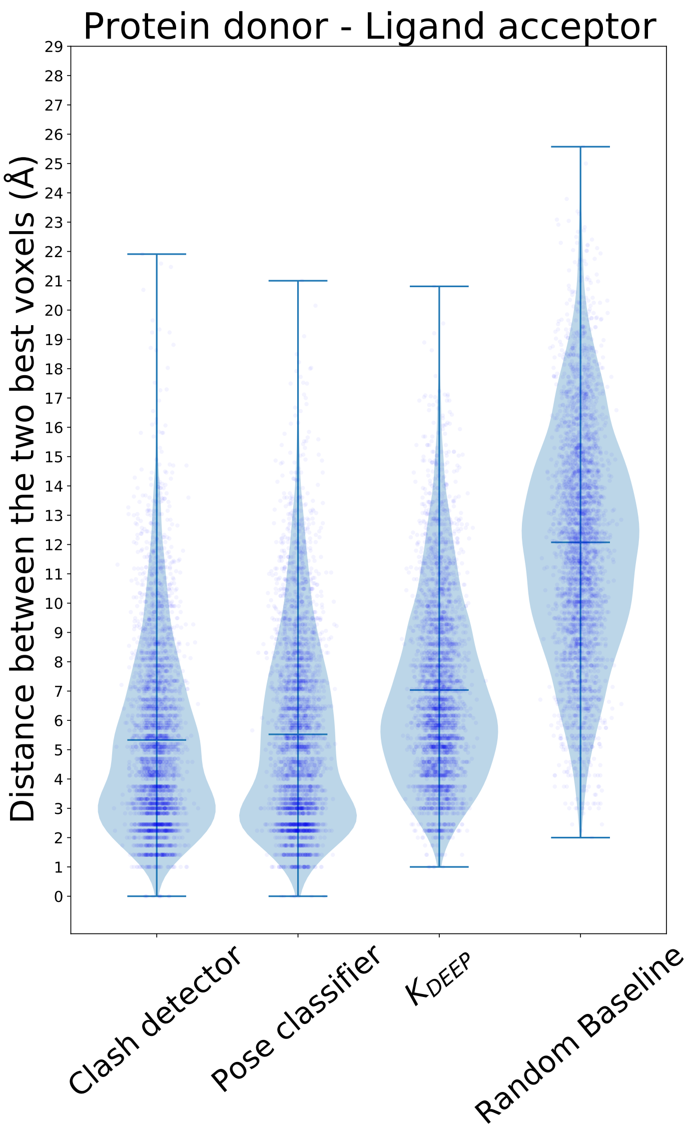

Figure S4: Distance distribution between the two voxels with highest, absolute attribution value in protein donor and ligand acceptor channels.

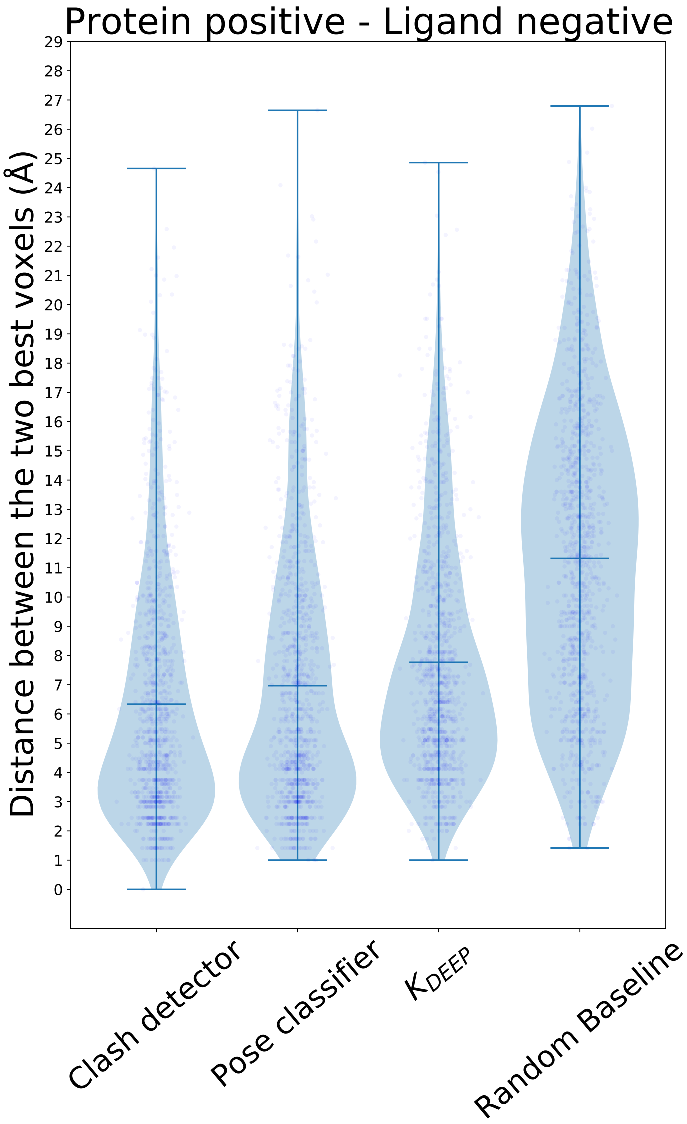

Figure S5: Distance distribution between the two voxels with highest, absolute attribution value in protein positive and ligand negative channels.

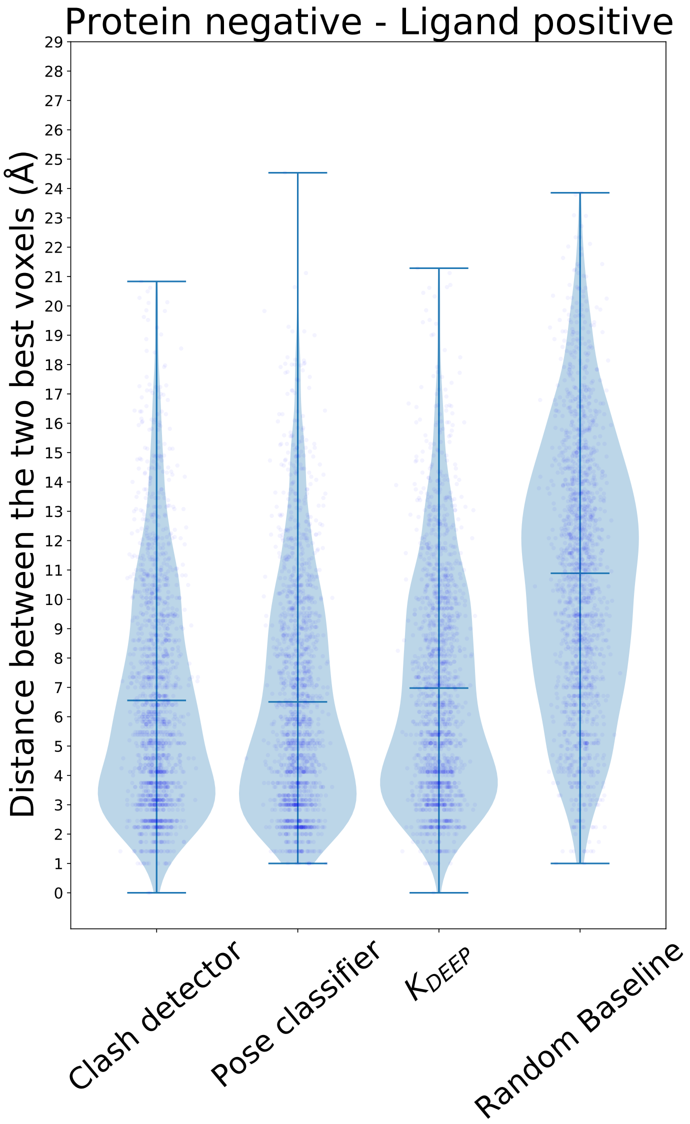

Figure S6: Distance distribution between the two voxels with highest, absolute attribution value in protein negative and ligand positive channels.

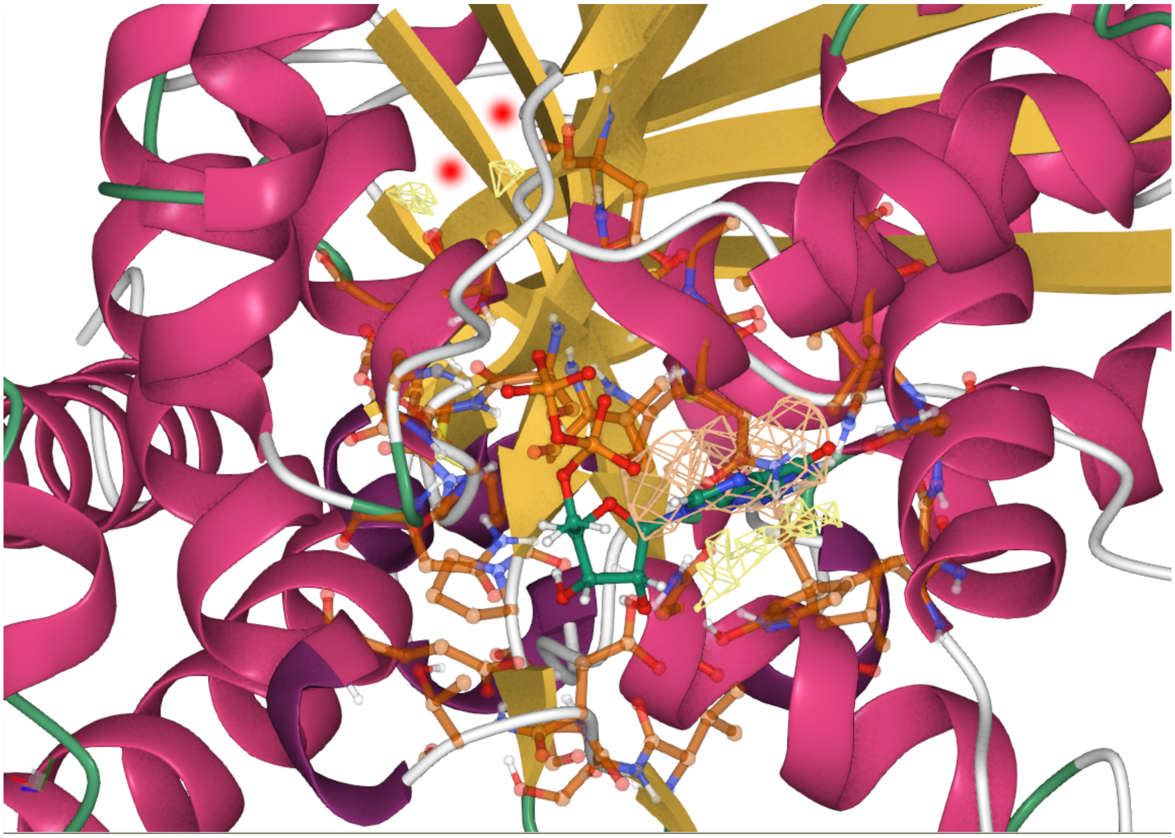

Figure S7: Attributions computed for the pose classifier model for the protein and ligand aromatic channels (yellow and brown, respectively). In addition to highlighting the aromatic ring in the Tyr residue engaging in a pi-stacking interaction with the ligand (lower right corner), two other regions in the aromatic protein channel appear highlighted (red dots at the top), despite being far apart from the ligand. A His residue can be found at that location (not shown for clarity) but no interaction with the ligand is possible as a beta sheet sits between the two parties. PDB code: 5JVD

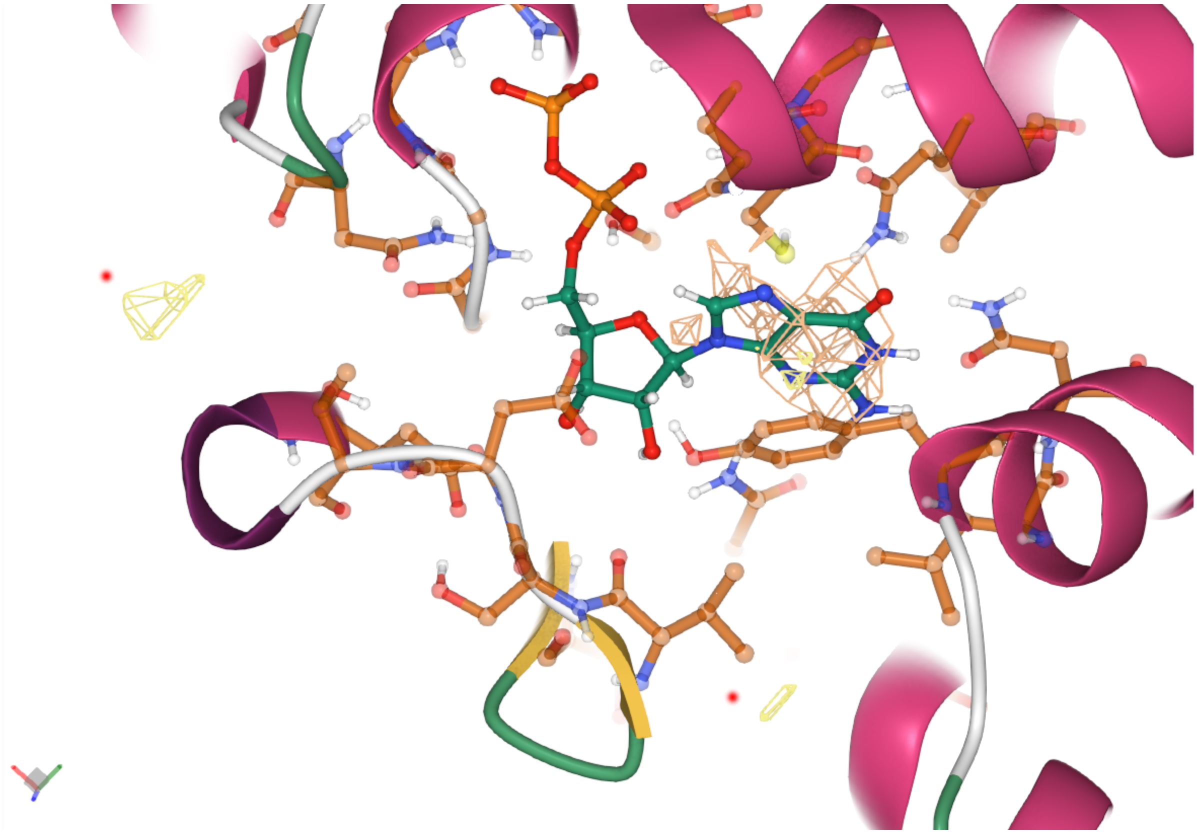

Figure S8: Attributions computed for K_{DEEP} model for the protein and ligand aromatic channels (yellow and brown, respectively). In addition to highlighting the aromatic ring in the Tyr residue engaging in a pi-stacking interaction with the ligand, two extra regions appear highlighted in the protein aromatic channel (identified by two red dots), despite being far apart from the ligand. The aromatic residues present at those locations (not shown for clarity) are a Trp (left red dot) and a Tyr (bottom right dot). Both are far away and shielded from the ligand by other protein residues. PDB code: 5JVD

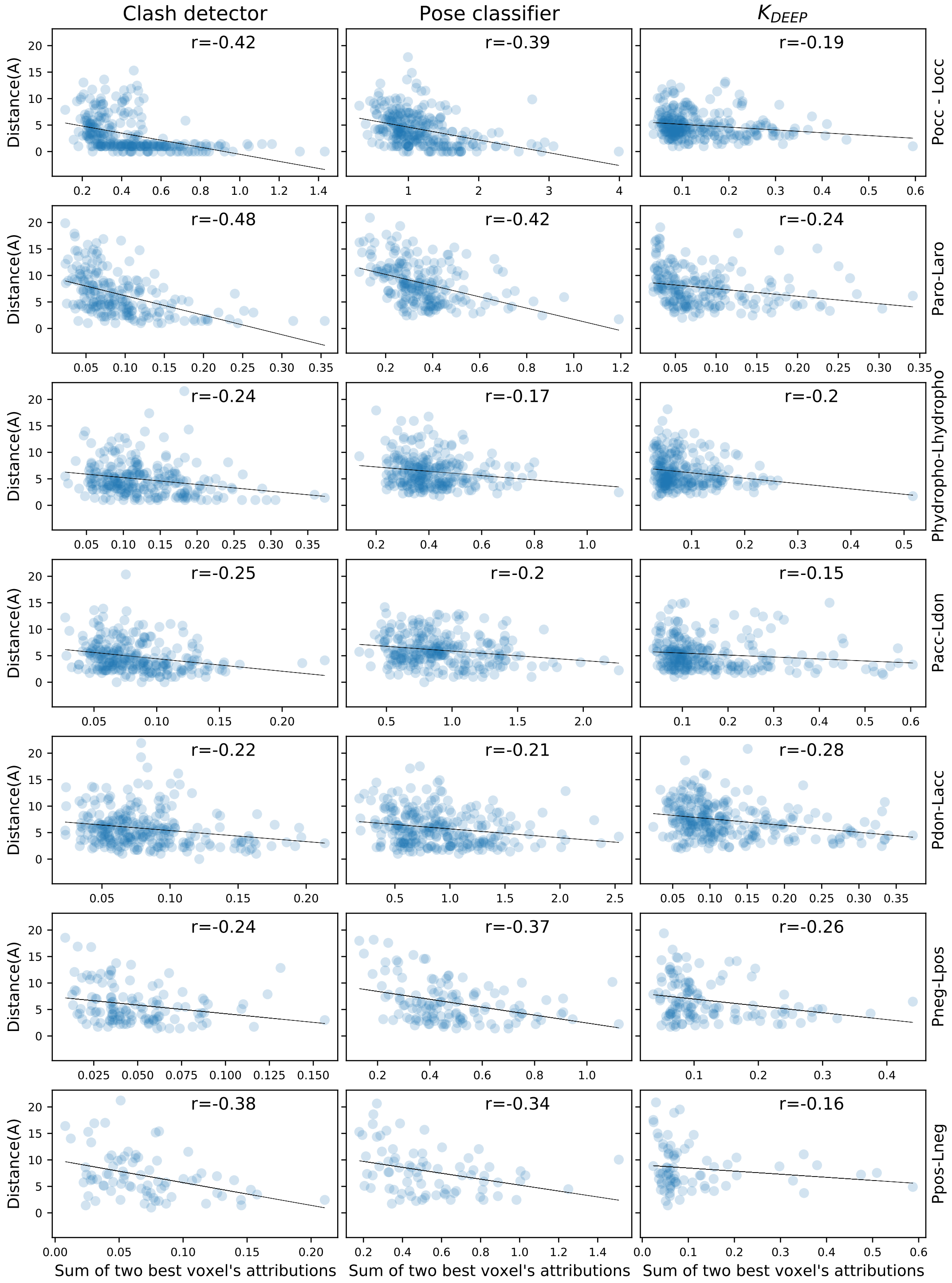

Figure S9: Correlation between the sum of the attributions of the two best voxels and distance between them. Each column represents a model and each row represents a protein-ligand channel combination (P is for protein, L for ligand, occ is occupancy, aro is aromatic, hydropho is hydrophobic, acc is acceptor, don is donor, neg is negative and pos is positive.)

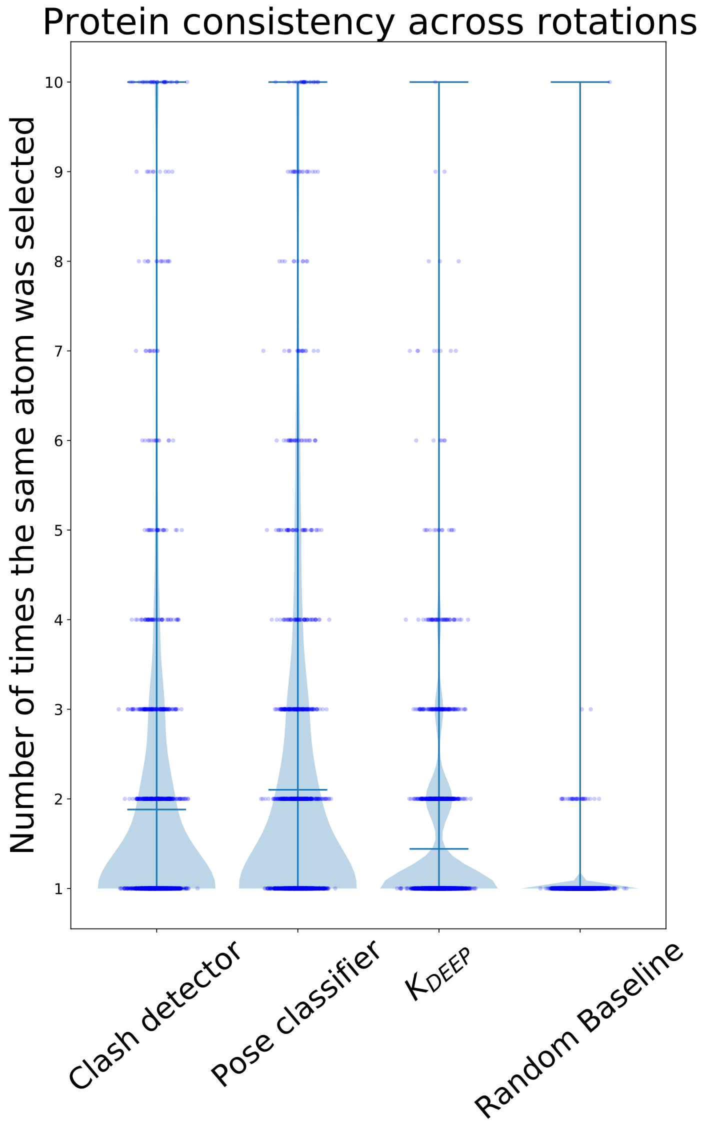

Figure S10: Consistency in protein atom attributions across 10 different orientations. The protein atom closest to the best voxel in the protein occupancy channel is identified in each of the 10 orientations. For each complex, we plot how many times the same atom was selected. As can be seen, in the clash detector and pose classifier models, for some complexes, the exact same protein atom is picked in all 10 rotations. In all three models, the distribution is clearly shifted upwards (more consistent) compared to the random baseline.

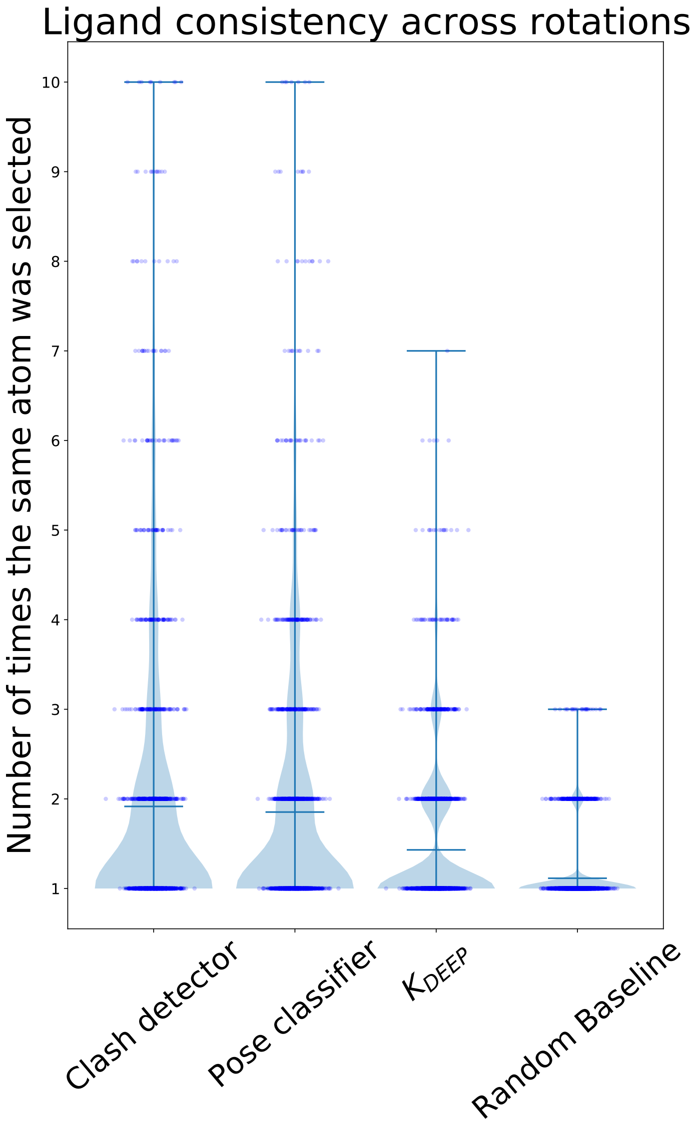

Figure S11: Consistency in ligand atom attributions across 10 different orientations. The ligand atom closest to the best voxel in the ligand occupancy channel is identified in each of the 10 orientations. For each complex, we plot how many times the same ligand atom was selected. All three models show a distribution more shifted towards greater values than the random baseline.

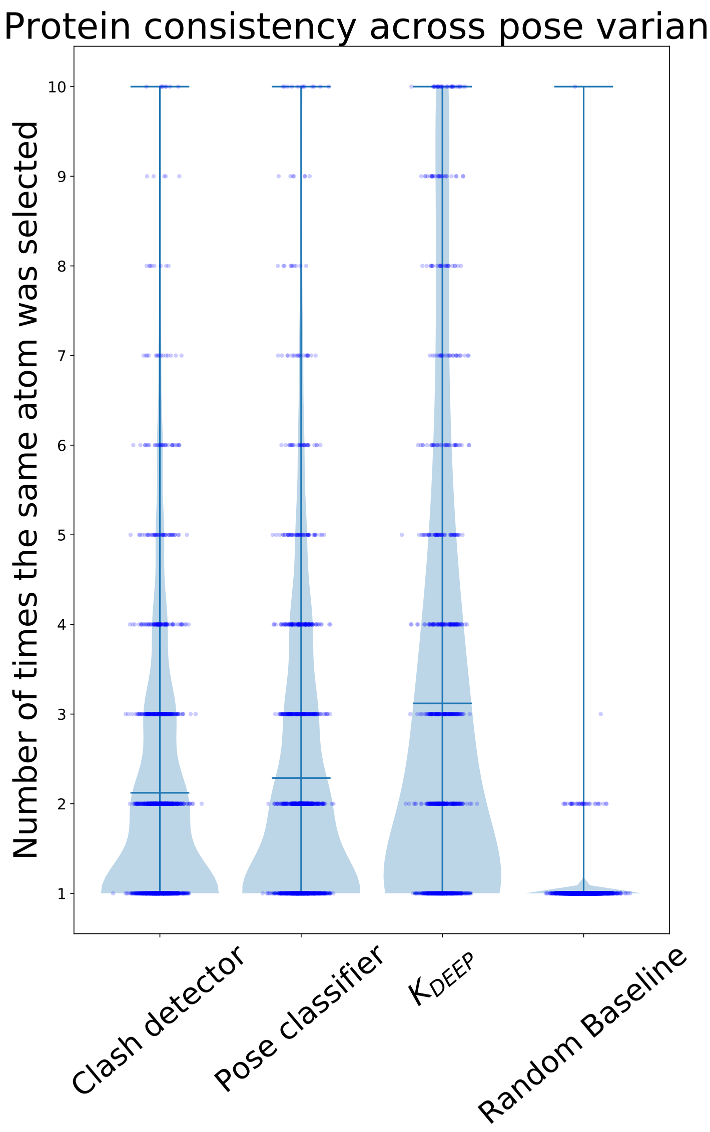

Figure S12: Consistency in protein atom attributions across 10 pose variations. The protein atom closest to the best voxel in the protein occupancy channel is identified in each of the 10 variants. For each complex, we plot how many times the same atom was selected. In K_{DEEP}, the exact same protein atom is select in all 10 pose variants for a large number of complexes.

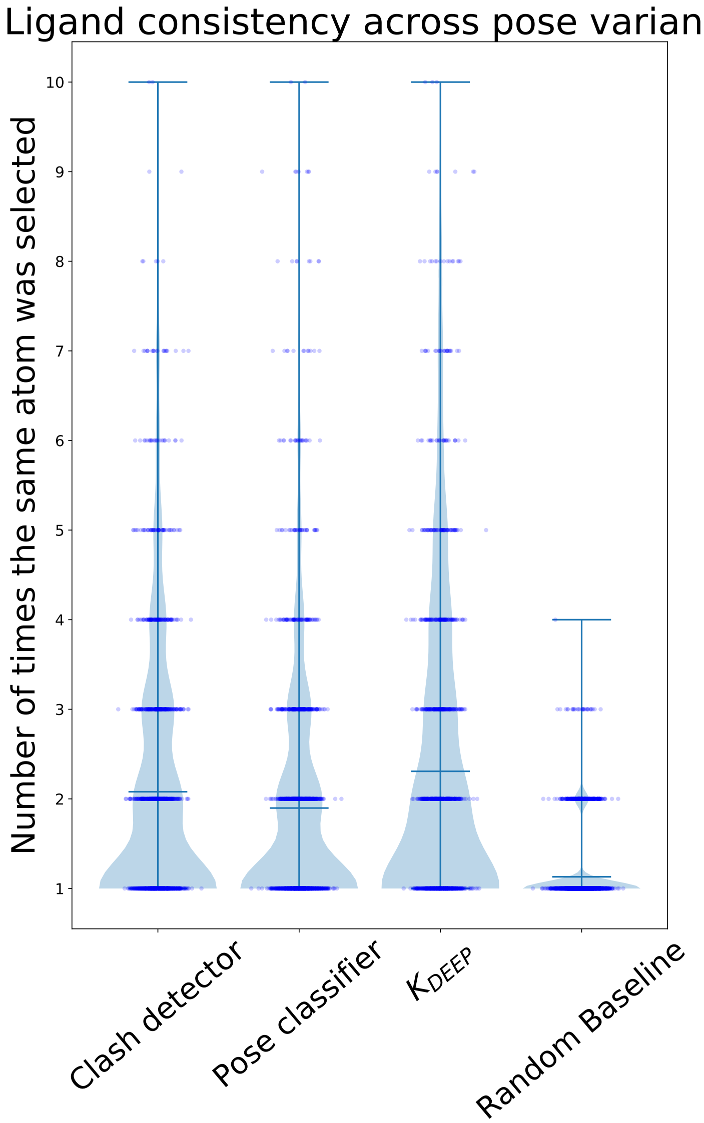

Figure S13: Consistency in ligand atom attributions across 10 pose variations. The ligand atom closest to the best voxel in the ligand occupancy channel is identified in each of the 10 poses. For each complex, we plot how many times the same ligand atom was selected. Although all three models show a distribution better than the baseline, unfortunately, there are few complexes on which the exact same ligand atom is picked in all 10 poses.

References

(1) Liu, Z.; Li, Y.; Han, L.; Li, J.; Liu, J.; Zhao, Z.; Nie, W.; Liu, Y.; Wang, R. PDB-wide collection of binding data: current status of the PDBbind database. *Bioinformatics* **2015**, *31*, 405–412.

(2) Ragoza, M.; Hochuli, J.; Idrobo, E.; Sunseri, J.; Koes, D. R. Protein-Ligand Scoring with Convolutional Neural Networks. *J. Chem. Inf. Model.* **2017**, *57*, 942–957.

(3) BCEWithLogitsLoss — PyTorch 1.8.1 documentation. `https://pytorch.org/docs/stable/generated/torch.nn.BCEWithLogitsLoss.html`.

(4) Hu, L.; Benson, M. L.; Smith, R. D.; Lerner, M. G.; Carlson, H. A. Binding MOAD (Mother of All Databases). *Proteins: Struct., Funct., Genet.* **2005**, *60*, 333–340.

(5) Ruiz-Carmona, S.; Alvarez-Garcia, D.; Foloppe, N.; Garmendia-Doval, A. B.; Juhos, S.; Schmidtke, P.; Barril, X.; Hubbard, R. E.; Morley, S. D. rDock: A Fast, Versatile and Open Source Program for Docking Ligands to Proteins and Nucleic Acids. *PLoS Comput. Biol.* **2014**, *10*, e1003571.
